# Description and potential sources of a shell deformity in North American freshwater mussels (Unionoida)

**DOI:** 10.1002/aah.10232

**Published:** 2024-12-01

**Authors:** Peter D. Hazelton, Andrew Gascho Landis, Andrew McElwain, Kyle Olivencia, Jason Carmignani

**Affiliations:** ^1^ D. B. Warnell School of Forestry and Natural Resources University of Georgia Athens Georgia USA; ^2^ Department of Fisheries, Wildlife, and Environmental Science State University of New York at Cobleskill Cobleskill New York USA; ^3^ Department of Biological Sciences State University of New York at Oswego Oswego New York USA; ^4^ Florida Fish and Wildlife Conservation Commission Fish and Wildlife Research Institute Gainesville Florida USA; ^5^ Massachusetts Division of Fisheries and Wildlife Natural Heritage and Endangered Species Program Westborough Massachusetts USA

**Keywords:** annuli, Chironomidae, parasite, shell deformity, Unionicolidae, Unionidae, *Xenochironomus*

## Abstract

**Objective:**

Freshwater mussels of the order Unionoida are among the most imperiled taxa in North America, and many species are undergoing enigmatic decline without fully understood causation. Disease pathology and parasitology have been identified as areas with significant knowledge gaps in relation to these declines. We investigated a shell deformity of unknown cause that is widespread in northeastern North America by adding to the clinical description from a mussel assemblage in Massachusetts with a deformity prevalence exceeding 50%. We build upon previous qualitative descriptions of this deformity with investigations of shell morphology and mussel age.

**Methods:**

We conducted a qualitative survey of the mussel community to evaluate the prevalence of deformity. Mussels were classified as deformed based on the presence of a distinct truncation of the posterior margin of the shell. For the eastern elliptio *Elliptio complanata*, we evaluated the shell height, shell length, and height : length ratio of animals classified as deformed versus normal and we conducted a comparison to a reference population. We also incorporated shell thin sectioning and aging to qualitatively describe the deformity in cross section and to compare age distributions between deformed and normal eastern elliptio.

**Result:**

We observed the presence of this deformity in four species, including the eastern elliptio, eastern lampmussel *Lampsilis radiata*, eastern pearlshell *Margaritifera margaritifera*, and creeper *Strophitus undulatus*. In cross section, the deformity appeared to be caused by repeated disturbance in growth in the posterior portion of the shell. Deformed eastern elliptio had markedly shorter shells for a given shell height when compared to normal and reference mussels, and they tended to be older at shorter shell lengths than normal mussels from the same site.

**Conclusion:**

The cause of the shell deformity in the United States remains unknown, although it appears similar in description to the deformity caused by a commensal midge, *Xenochironomus canterburyensis*, which infects a distantly related freshwater mussel in New Zealand. We highlight potential causes and the need for further investigation.

## Abstract 2

**Impact Statement**

Freshwater mussels in the order Unionoida are among the most imperiled taxa in North America; pathogens, parasites, and environmental contaminants are among several potential causes of endangerment. Our work adds to the description of a deformity observed in mussels from northeastern North America and outlines a potential parasitic cause evidenced from across the globe.

## INTRODUCTION

Freshwater mussels (order Unionoida) are among the most imperiled faunal groups in North America (Haag and Williams 2014). Drastic declines in freshwater mussel populations disrupt the ecosystem services provided to aquatic ecosystems, including substrate stability (Allen and Vaughn 2011), filtration (Vanden Byllaardt and Ackerman 2014), nutrient cycling (Atkinson and Forshay 2022), and food web dynamics (Atkinson et al. 2021). Despite the high levels of imperilment, relatively little is known about the influence of disease and parasites on freshwater mussel population dynamics and species declines (McElwain 2019; Aldridge et al. 2023; Knowles et al. 2023). There has been a recent influx of research that explores the health of freshwater mussels by examining the role of viruses (Richard et al. 2020, 2021), bacteria (Leis et al. 2023a, 2023b), and parasites (Brian and Aldridge 2019; Brian et al. 2021; Knowles et al. 2022) as potential causes of population declines.

Along with growing interest and the need to understand the causes and consequences of unionid declines, evaluation of shell abnormalities has been proposed as a research area warranting further investigation (McElwain 2019; Knowles et al. 2023). Shell abnormalities and deformities vary in presentation and are associated with a variety of potential causes, including mechanical injury, shell erosion and dissolution from low pH, mineralization, pearl formation, nacre discoloration, pustule formation, shell twisting, and shell truncation (reviewed by McElwain 2019; Knowles et al. 2023). Indeed, shell anomalies have been reported since the late 1800s (Beecher 1883; Baker 1901; Coker et al. 1921), a time during which mussel shells held considerable value in the pearl and button industries (Coker et al. 1921; Haag 2012).

Strayer (2008) observed a consistent shell deformity over a 10‐year period among five unionid species in five New York rivers (Strayer 2008). The deformity was described as having “irregular or ragged” posterior ends, and Strayer (2008) further stated that “the periostracal layers often are thick and distorted” and that “the [two] valves often do not fit together well at the posterior end of the shell, resulting in a marked gap.” The severity of the shell deformity sometimes reached a level that caused difficulty in identifying the species. The proportion of deformed individuals was over 10% in all populations. Parasitic infection and chemical toxicity were hypothesized as possible causes of the deformity, but no further evaluation was conducted.

Parasites and commensal organisms have long been suspected to cause shell anomalies in freshwater mussels (Coker et al. 1921; Howells et al. 1996; Parmalee and Bogan 1998; McElwain 2019). Among those are blister pearls that form in the pallial cavity, where the nacreous layer of the shell is laid down over the top of foreign material, including occasional parasites (McElwain 2019). Prior to the study by Strayer (2008), there were only a few North American studies that referred to shell margin distortion, shortening, and thickening attributed to the presence of parasites. Most notably, Howells et al. (1996) reported a shell that was most similar to the deformity reported by Strayer (2008). Coker et al. (1921) reported deformities generated by parasites in the anterior margin of the shell, causing thickening and shortening of the shell. However, neither Coker et al. (1921) nor Howells et al. (1996) described the parasite responsible for the deformity.

Although a parasite causing shell truncation has not been identified from North America, the larvae of the midge *Xenochironomus canterburyensis* (order Diptera, family Chironomidae) have been described as parasites of a freshwater mussel, the kākahi *Echyridella menziesii* (formerly *Hyridella menziesi*; Hyriidae), in New Zealand (Forsyth and McCallum 1978; also see Graf and Cummings 2021). The larvae reside between the shell and the mantle tissue until the third or fourth instar (Forsyth 1979) and may disrupt the development of the prismatic and periostracal layers, resulting in a truncation of the posterior region of the shell (Forsyth and McCallum 1978). In two lakes in New Zealand, over 50% of mussels captured in surveys had internal shell abnormalities, including deformities at the posterior shell margin (Roper and Hickey 1994). The deformities were suggested to be related to the presence of *X. canterburyensis* larvae that were observed in the shells.

In 2017, an assemblage of freshwater mussels with deformed shells was identified in the North Nashua River, Massachusetts. The deformity closely matched the descriptions by Strayer (2008) and others (Forsyth and McCallum 1978; Forsyth 1979). The goal of our study was to further characterize the shell deformity and explore the influence on mussel populations by identifying the species impacted and the deformity's frequency and severity; comparing shell morphometrics between deformed and normal mussels from the North Nashua River and mussels from a reference population; and comparing length and ages of deformed and normal mussels. Further investigation into this deformity will provide insight into the impact that the deformity may have on the health of these mussel populations.

## METHODS

### Site description

**FIGURE 1:**
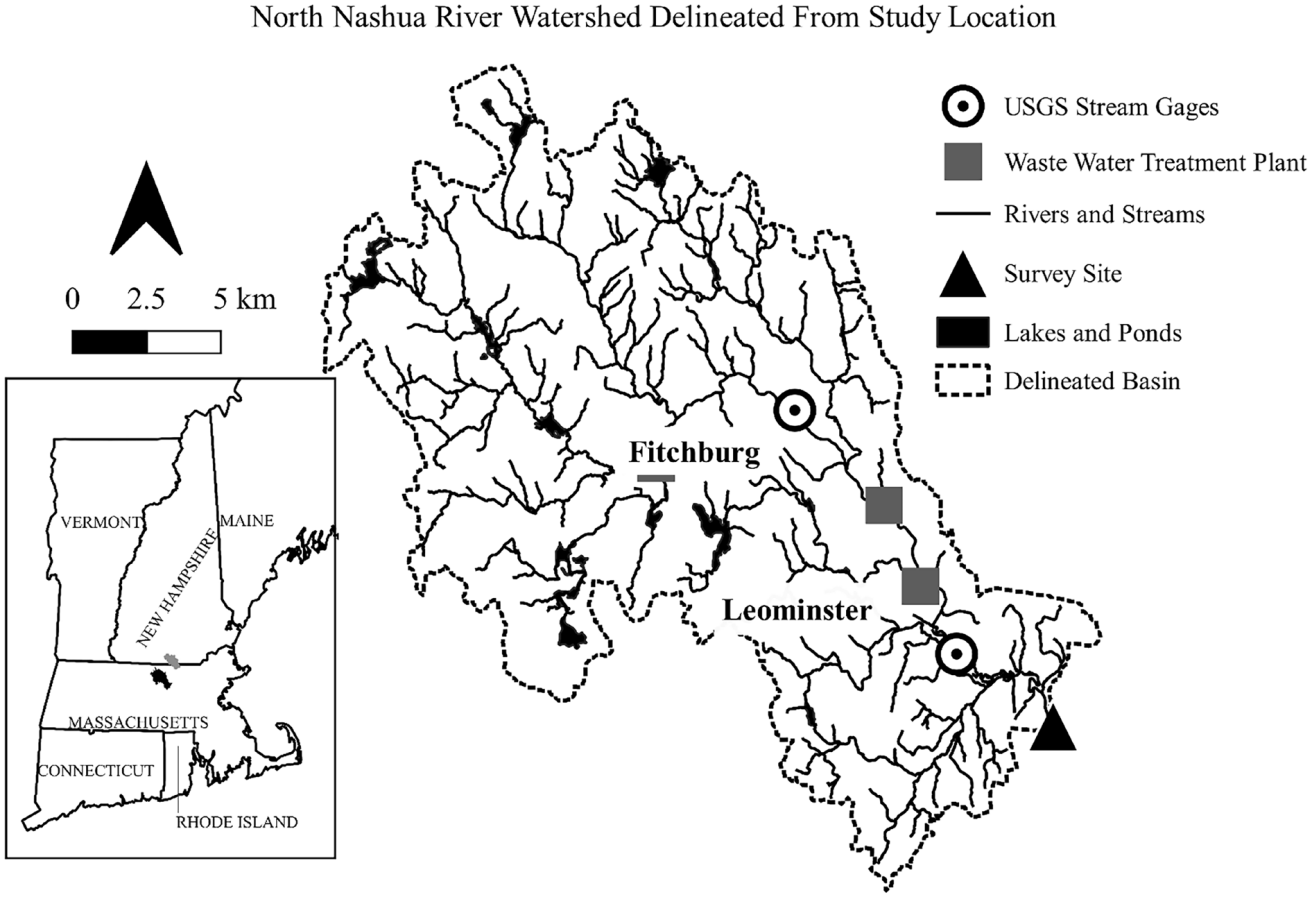
The North Nashua River watershed, Massachusetts, delineated from the study site, including the locations of wastewater treatment sites, reservoirs and lakes, and U.S. Geological Survey (USGS) stream gauges. Inset depicts the location of the study area (black) and the reference watershed (gray) within the New England states, northeastern United States.

The North Nashua River is a 60.4‐km (37.5‐mi) long, fourth‐order river. Its watershed is situated in north‐central Massachusetts and southeastern New Hampshire, covering 1393.41 km^2^ (538 mi^2^; Figure 1). According to the 2011 National Land Cover Dataset (Multi‐Resolution Land Characteristics Consortium [MRLC] 2011), the basin is approximately 57.47% forested, 25.0% urban developed, and 10.6% impervious surface (U.S. Geological Survey 2019). The site of mussel collection was in Lancaster, Massachusetts, downstream of the cities of Fitchburg (population 41,946; U.S. Census Bureau 2020) and Leominster (population 43,728; U.S. Census Bureau 2020), Massachusetts, and parts of surrounding towns. Both Fitchburg and Leominster have a rich history of industrial manufacturing. Over 32 km (20 mi) of the river are registered under the U.S. Clean Water Act 303(d) list of impaired water bodies (Massachusetts Department of Environmental Protection 2008), and the study site receives waters that include most of the wastewater effluent from Fitchburg and Leominster.

To compare mussel shell morphology characteristics to those of a reference population, we used shell data from the Nissitissit River in Pepperell, Massachusetts, which were available in the Massachusetts Division of Fisheries and Wildlife (MDFW) mussel databases. The Nissitissit River is a third‐order tributary stream of the North Nashua River and is located approximately 50 km (31 mi) downstream from the survey site. The watershed feeding the Nissitissit River (157‐km^2^ watershed size; 172 km of streams) is smaller than that of the survey site. The Nissitissit River watershed also has markedly different land use than the North Nashua River (78.11% forested, 7.28% urban development, and 1.72% impervious surface; MRLC 2011). Differences in the land uses between these sites, as well as the similarity in the mussel assemblages represented across sites, are valuable for contrasting differences in the shell morphology of mussels from these streams. We used data from mussels at the reference site only in comparison to shell morphology at the affected stream. No age data were generated for mussels from the reference site.

### Field collection and shell measurement

Mussels were collected from the North Nashua River site on August 12, 2017, during a qualitative, unrestricted survey. Depth was less than 1 m in the center of the stream, and a single observer worked both banks and surveyed the center of the stream. Full coverage was not possible, and the observer meandered across bank and stream center substrates in an upstream direction to evaluate representative habitat. Approximately 50 m of stream were surveyed, and all mussels and shells observed were collected for evaluation.

**FIGURE 2:**
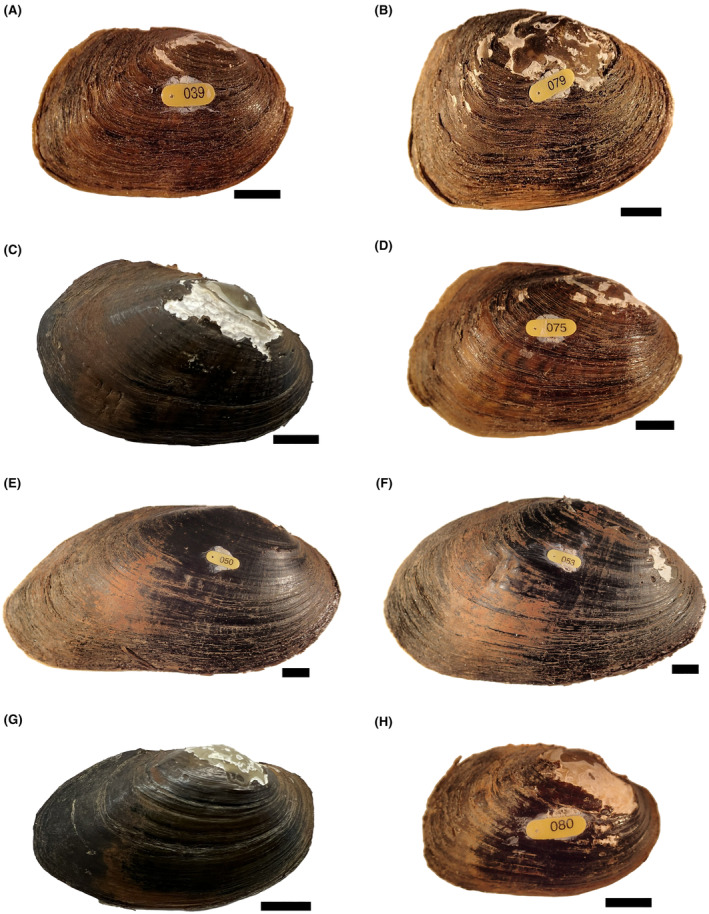
Comparative presentation of normal (left) and deformed (right) shells across species: (A), (B) eastern elliptio; (C), (D) eastern lampmussel; (E), (F) eastern pearlshell; and (G), (H) creeper. All specimens were collected at the North Nashua River, Massachusetts, study site except as follows: the eastern lampmussel in panel C was collected from the Taunton River, Plymouth County, Massachusetts; and the creeper in panel G was collected from the Mill River, Franklin County, Massachusetts. (Scale bars = 1 cm).

The collected living mussels and shells were brought back to the MDFW laboratory in Westborough, Massachusetts, where shell measurements were made, including length and height to the nearest 0.1 mm. A total living wet weight (whole animal and shell) was recorded to the nearest 0.1 g. All mussels and shells were tagged with a uniquely identifying shellfish tag (Floy Tag, Inc.) and cyanoacrylate glue for further identification during initial shell measurements and thin sectioning. A single observer (P. D. Hazelton) assigned mussels to either deformed or normal classes by visual inspection. Normal mussels had no signs of truncation in the posterior end, whereas deformed animals were truncated and the posterior margin of the shell was growing in toward the center line of the mussel body (Figure 2). The prevalence of deformity was calculated as the percentage of individuals with deformity for each species in proportion to the total number of animals collected at the site.

### Shell sectioning and aging technique

**FIGURE 3:**
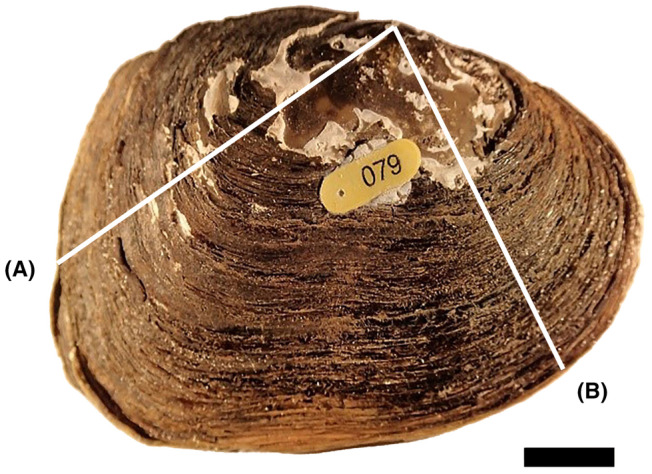
Diagram of an eastern elliptio, showing the location of thin section cuts for visual inspection of the deformity growth pattern (A) and the thin section for aging (B). (Scale bar = 1 cm).

Thin sectioning and shell aging analyses were conducted at the Department of Fisheries, Wildlife, and Environmental Science, State University of New York (SUNY) at Cobleskill. Sections were cut from the posterior end of several specimens with and without deformity to qualitatively evaluate and describe the effect of the deformity on shell structure (Figure 3), whereas a section taken from the anterior of the shell through the umbo was used for shell aging (Figure 3). Section cuts were made using a low‐speed IsoMet diamond saw (Buehler) by starting at the umbo and moving outward toward the farthest anterior or posterior point of the shell. Polishing was done on each cut shell by using wet sandpaper in increasing fineness levels of 320, 400, 600, 1000, 1500, 2000, and 2500 grit. We rinsed shells in between switching grits and after the final polish. Shells were left to dry for 1–2 h before mounting. To mount the freshly polished shells onto a glass slide, we applied a two‐part epoxy to the polished side of the shell and then pressed it onto the slide. The slides were dried overnight before we mounted them to a wafer chuck. Mounting to the wafer chuck was done by heating the chuck, melting paraffin wax onto it, and then firmly pressing the slide onto the melted wax. Excess wax was removed, and the chuck was then mounted to the arm of the saw. The saw was set to cut a thin section width of approximately 0.75 mm starting from the anterior portion to the umbo, opposite of the initial cut. After we finished cutting the thin sections, we removed the chucks and polished the thin sections once more according to the previous procedure.

To age the thin sections, we counted the internal growth annuli of the shell because it is recommended as the most accurate form of aging freshwater mussels (Haag and Commens‐Carson 2008; Dycus et al. 2015). Using a dissecting microscope, two individuals counted and recorded the number of opaque growth lines occurring on each individual thin section. After all shells were aged, the final ages were compared. If different ages were given for an individual, the shell was reanalyzed until both readers came to an agreement on the most accurate age. If a consensus could not be reached, the shell was removed from the study.

### Comparison of shell morphology and age

We compared shell measurements between deformed mussels, normal mussels (i.e., mussels without deformities), and mussels from a reference population when enough representatives of a species were collected (Table 1). We did not conduct hypothesis‐based statistical comparisons between mussel groups because of the nature of our sampling at the site with the deformity. First, timed visual surveys of freshwater mussels are known to be biased toward collection of larger individuals and do not adequately represent the abundance or size distribution of the population (Strayer and Smith 2003). Second, the survey methods used at the study site differed from the methods used to collect data at the reference site and therefore cannot be considered a one‐to‐one comparison. Finally, the determination of deformity was conducted via gross visual inspection and was not independent of shell morphological features. Nevertheless, we provide qualitative descriptive comparisons of shell morphology and age between mussel groups, as these aid in our objective to better define the shell deformity, including differences in shell height, shell length, height : length (H:L) ratio, and age between evaluated groups. Summary statistics were calculated using R version 4.3.1 (R Core Team 2023), and plots were generated using the ggplot2 package (Wickham 2016).

**TABLE 1:** Shell length range (mm) and number of individuals (*n*; in parentheses) per species in each deformity class at the North Nashua River, Massachusetts, study site and the Nissitissit River reference site. Percent deformed was calculated as the percentage of deformed individuals at the North Nashua River site. Reference population information is presented only for the species with adequate abundances for comparisons between sites.

Species	Reference site	Study site		
		Normal	Deformed	Percent deformed
Eastern elliptio *Elliptio complanata*	25–82 (51)	26.7–99 (36)	42–91.1 (36)	50
Eastern lampmussel *Lampsilis radiata*	–	49.6–53.3 (2)	68.8 (1)	33
Eastern pearlshell *Margaritifera margaritifera*	–	86–119.5 (3)	97.7–113.8 (4)	57
Creeper *Strophitus undulatus*	–	–	48.1 (1)	100

## RESULTS

**FIGURE 4:**
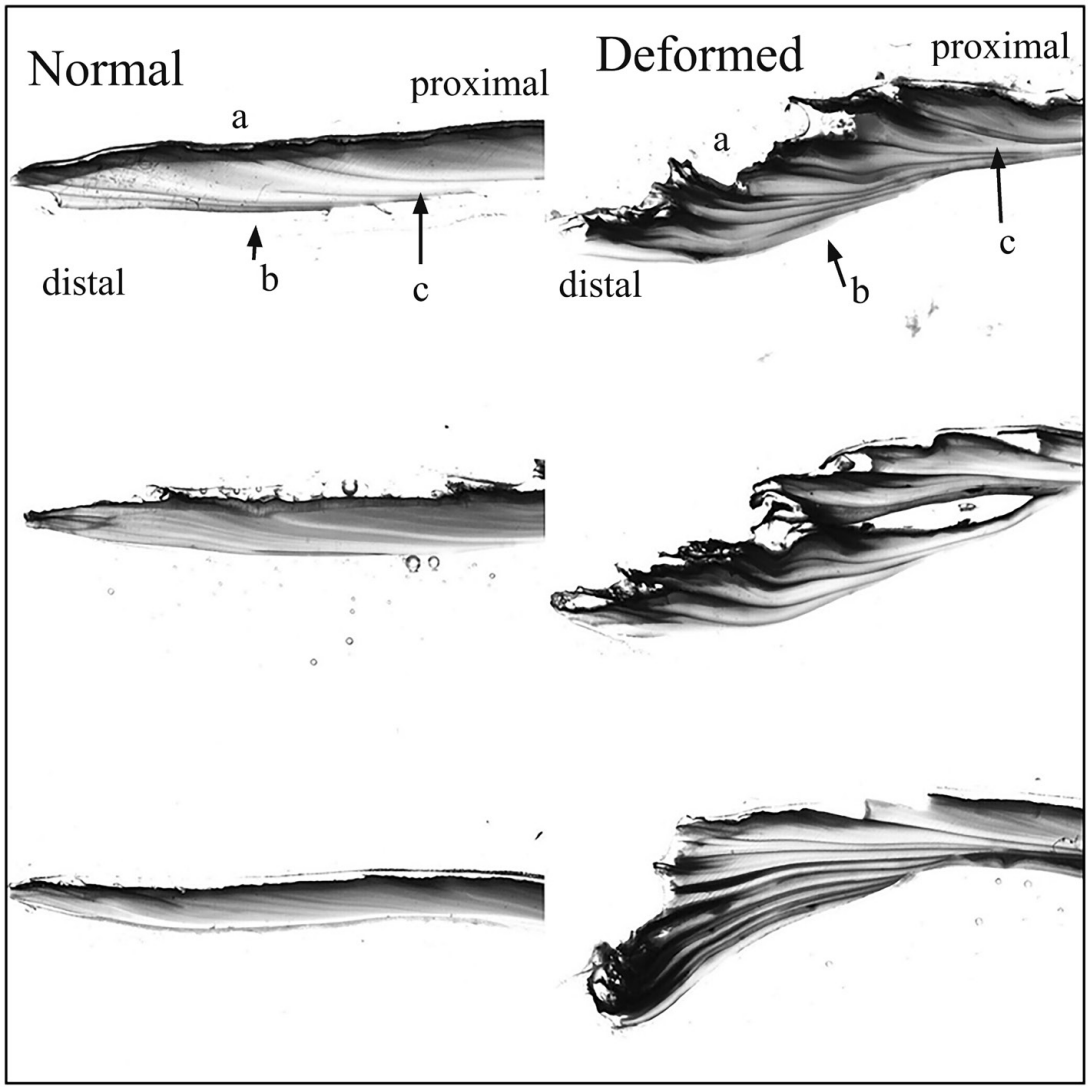
Comparison of shell thin sections from normal (left) and deformed (right) eastern elliptio. Each picture represents a thin section from one individual. Sections were made through the umbo (proximal) to the posterior‐ventral (distal) margin of the shell, showing the periostracum (a), the nacreous surface (b), and the growth rings (c). Deformed mussels were characterized by what appears to be a repeatedly broken or disturbed trajectory of growth, resulting in a ragged periostracum and possible disturbance rings.

We recorded deformed individuals of all four mussel species observed at the North Nashua River site (Table 1). Observed abundances ranged from a single deformed creeper to 72 deformed eastern elliptio. In all four species, the deformity presented as a truncation of the posterior edge of both valves (Figure 2) such that the valve appeared to be growing inward. A gradient of severity appeared to occur within deformed shells, particularly those of eastern elliptio (Figure S1); however, we were unable to identify a discrete threshold for these severity classes, so we report animals here as either “deformed” or “normal.” In cross section, the shell appeared to be thickening as it was growing inward, and disturbance rings appeared thickened, fractured, or misaligned (Figure 4). The periostracum also became very rough in deformed individuals. The presentation was qualitatively different across species and was more apparent in smaller species at our site (i.e., eastern elliptio and creeper) than in eastern pearlshell (Figure 2).

**FIGURE 5:**
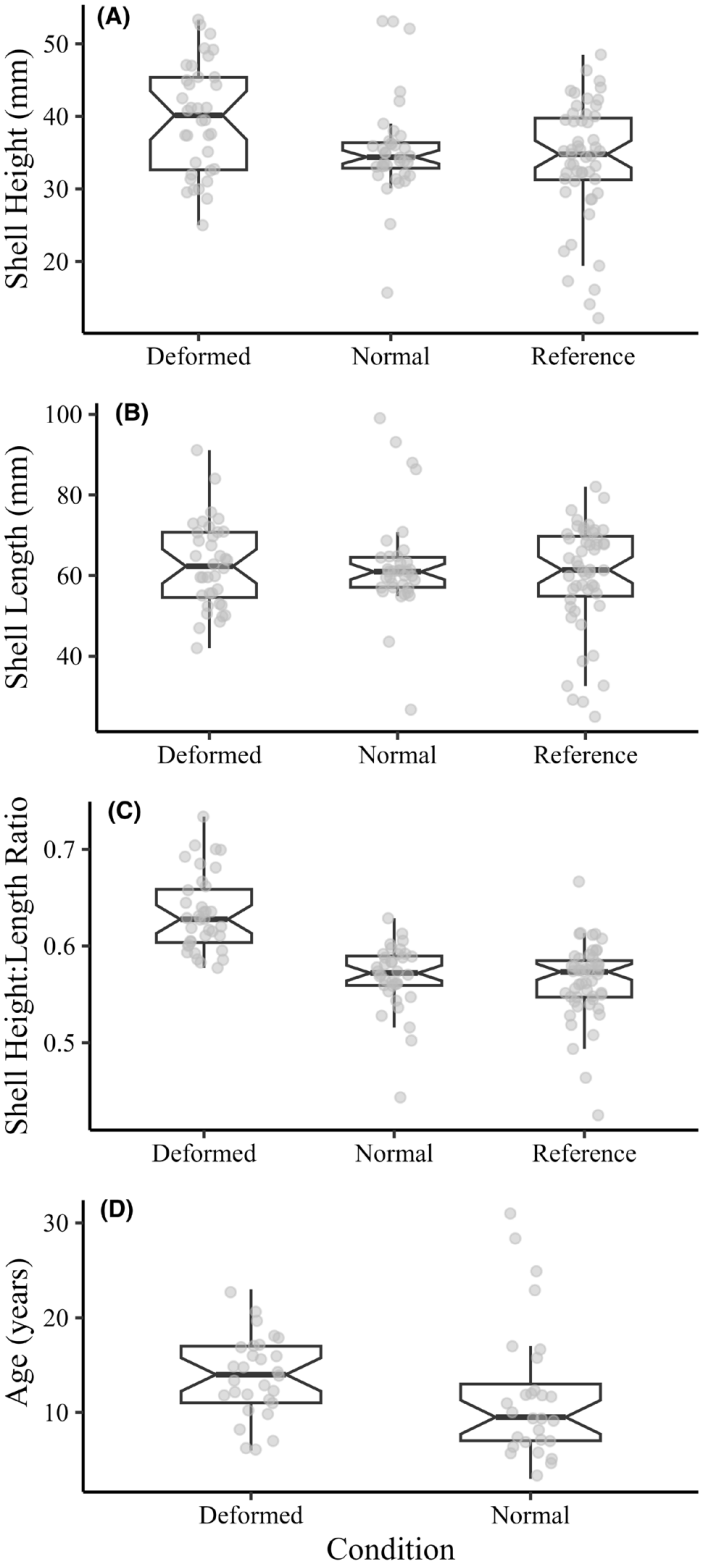
Box‐and‐whisker plots displaying morphological and age comparisons between deformed, normal, and reference eastern elliptio, including differences in (A) shell height (mm), (B) shell length (mm), (C) shell height : length ratio, and (D) ages (years) estimated from thin sectioning and annulus counting. For each box plot, the horizontal black bar represents the median value; the extent of the box represents the interquartile range (IQR); whiskers represent 1.5 × the IQR; gray circles represent individual data points; and notches within each box represent the 95% confidence interval around the median.

Only the eastern elliptio was abundant enough at the North Nashua River site (*n* = 72) for a comparison of morphology and age across deformity condition (i.e., deformed vs. normal) and for comparison to the reference population in the Nissitissit River (*n* = 51; morphological comparison only). Qualitative comparisons of the data are represented in Figure 5 and are discussed briefly here. Central tendencies of shell heights were highest among deformed mussels (*n* = 36; mean = 39.7 mm; median ± 95% confidence interval [CI] = 40.2 ± 3.36 mm) when compared to mussels identified as normal (*n* = 36; mean = 35.5 mm; median ± 95% CI = 34.4 ± 0.93 mm) and those from a reference population (*n* = 51; mean = 33.9 mm; median ± 95% CI = 34.8 ± 1.88 mm). Minimum shell heights were also higher in deformed mussels (25 mm) than in normal (15.7 mm) or reference (12.2 mm) mussels. Maximum shell heights were not as markedly different between groups (Figure 5A).

Shell lengths were not distinctively different among deformed, normal, and reference eastern elliptio (Figure 5B), but minimum shell lengths were markedly smaller in normal (26.7 mm) and reference (25 mm) populations than in deformed mussels (42 mm). Additionally, maximum shell lengths were greater in mussels identified as normal (99 mm) than in those identified as deformed (91.1 mm) or in mussels from the reference population (82 mm). Conversely, the H:L ratio showed the greatest differences between deformed mussels and the mussels identified as normal or mussels from the reference population (Figure 5C). Consistently higher H:L ratios were found in deformed mussels (mean = 0.633; median ± 95% CI = 0.628 ± 0.015), whereas the H:L ratios were similar between normal mussels (mean = 0.569; median ± 95% CI = 0.572 ± 0.008) and reference mussels (mean = 0.564; median ± 95% CI = 0.573 ± 0.08). A stronger difference in the H:L ratio further supported that mussels classified as deformed had an identifiably different shape relative to normal or reference mussels, even though overall shell length and height differed less between the groups (Figure 5A,B).

Normal mussels from the primary study site showed a greater range of ages (*n* = 28; range = 3–31 years) compared to deformed mussels (*n* = 29; range = 6–23 years), despite central tendencies of age being greater in deformed mussels (Figure 5D). Although the four oldest mussels and the three youngest mussels evaluated were not classified as deformed, the median age of deformed mussels (median age ± 95% CI = 14 ± 1.76 years) was almost 5 years older than that of normal mussels (median age ± 95% CI = 9.5 ± 1.79 years). Thus, deformed mussels tended to be older than normal mussels even though the oldest mussels were not classified as deformed.

## DISCUSSION

Our results further describe a shell deformity in freshwater mussels that is rarely documented in North America. We documented this deformity in four species from a single location in Massachusetts. Currently, we cannot determine for certain that the deformity observed in our study is the same that Strayer (2008) described for mussels in New York State; however, combined with those previous results, the total number of documented North American mussel species that are susceptible to this deformity is eight species (representing seven genera and two families) across 10 sites in the northeastern United States.

The eastern elliptio was the only species in our sample for which we had enough observations to conduct formal morphological analysis of the deformity. We found that mussels with the deformity had an H:L ratio (Figure 5C) that was markedly different from those of mussels categorized as normal and mussels from the reference site, despite smaller differences between groups when the shell height (Figure 5A) or shell length (Figure 5B) was considered alone. The H:L ratio in normal mussels more closely matched that of the reference population (Figure 5C), whereas deformed mussels had distinctly taller shell heights at a given shell length.

Animal aging through thin sectioning revealed that deformed mussels were older on average than normal mussels (Figure 5D), despite the similarities in shell length between groups. Although deformed mussels were generally older than normal mussels, the oldest mussels at our study site showed no sign of deformity. It is possible that the deformity was caused by a discrete temporal exposure to mussels of a certain age; however, the overlap in age of deformed and normal mussels in this study would suggest that only certain individuals were either exposed or affected during that time. It is also important to note that our observations of age differences are based on only one eastern elliptio population that was sampled by using a qualitative timed survey. Such surveys are known to have a bias toward larger animals (Strayer and Smith 2003); therefore, assessment of the deformity in younger animals may not be feasible. Future studies targeting the age of deformity should incorporate quantitative mussel sampling methods to avoid sampling bias and to provide a better assessment of deformity onset at age.

In most cases, a mussel presenting the deformity appeared to have been affected continuously or repeatedly after an initial disturbance. This is evidenced by the shell cross sections of deformed animals in Figure 4. Therefore, it is difficult to ascertain whether the deformity was caused by (1) a repeated disturbance or (2) a single injury or infection, which permanently damaged the mantle and rendered the mussel unable to smoothly lay down shell. We also did not attempt to back‐calculate the specific year in which each deformed mussel first sustained its shell injury. Such data collected in the future may be helpful in establishing a time of exposure to the contaminants, pathogens, or parasites that are causing the deformity.

The hypothesis that contaminants are a cause of the deformity is intriguing, but the localized presentation of the deformity on the mussel's posterior would likely imply a toxicant with only a limited mode of action. Strayer (2008) proposed an environmental pollutant as one potential cause of the deformity because the collection sites in New York State were associated with agricultural land use or municipal wastewater. The North Nashua River has a long history of receiving municipal and industrial wastewater, and the river still receives effluent from three or more wastewater treatment plants (Figure 1). Although the potential for exposure to a xenobiotic is apparent, the site of action within the mussel is limited to the posterior end, suggesting a localized rather than systemic response. When a mussel is burrowing, the posterior margin of the shell, siphons, and mantle are most commonly in contact with the water column, while the rest of the animal may be burrowed in the sediment. There is a higher likelihood of exposure here, but shell growth occurs around the shell and mantle margin and the deformity is absent from the anterior and much of the central margin of the shell. If the toxicant had a systemic mode of action that interrupted sequestration and laying down of shell layers, we would expect the deformity to be present throughout the shell margin. The localized presentation of the deformity suggests that a toxicant is acting solely at the site of exposure, such as an irritant that may periodically disrupt shell growth at the site.

McElwain (2019) and Knowles et al. (2023) reviewed several known causes of shell deformities, including mechanical damage, shell erosion from friction or poor water quality, and the presence of parasites. We do not believe that mechanical damage to the shell (e.g., crushing) is a likely explanation for this truncation deformity, as the proportion of mussels affected is too great (e.g., ~50% of eastern elliptio) and the deformity is too consistent across individuals. Shell erosion also does not explain the truncation, as erosion and shell dissolution are typically observed as missing periostracum and erosion down into the prismatic layer of the shell (Roper and Hickey 1994; Haag 2012).

The deformity described in the current study and by Strayer (2008) appears most similar to that caused by *X. canterburyensis* in New Zealand (Forsyth and McCallum 1978). Posterior shell growth disruption was reportedly caused by *X. canterburyensis* larvae within the mantle margin, resulting in mechanical disturbance of growth along the prismatic and periostracal layers (Forsyth and McCallum 1978). Similar disruption of growth rings is well documented in mussels that have been marked along the shell margin with a file (i.e., shell notching; Haag and Commens‐Carson 2008), although the animals in our study appear to have had repeated growth disruptions (Figure 4).

Unionids are known to host a variety of parasites, including ciliates, trematodes, nematodes, mites, chironomids, and odonates (reviewed by Knowles et al. 2023). Some of these can cause considerable damage to gill and mantle tissue, but few resulting shell deformities have been described (Knowles et al. 2023). We did not observe any gross pathological changes to the mantle or other soft tissue during dissection. Histopathological evaluation found two eastern elliptio with mite eggs embedded in the posterior mantle edge, and the sole eastern lampmussel had a possible microsporidian infection. However, we did not see consistent signs of a parasite infection in affected mussels (A. McElwain, unpublished observations).

The absence of parasite stages during our sampling could be attributable to parasite phenology. Forsyth and McCallum (1978) found that later (third and fourth) instars of *X. canterburyensis* were found in kākahi mantle margins during the fall and winter, whereas earlier instars were only present in the pallial cavity during spring and summer. In North America, seasonal infestations of later instars of *Baeoctenus bicolor* and another chironomid on the gills of eastern floater *Pyganodon cataracta* (formerly *Anodonta cataracta*) and paper pondshell *Utterbackiana implicata* (formerly *A. implicata*) have been documented. Interestingly, these chironomids were only observed during late fall through spring (November–June; Gordon et al. 1978, reviewed by Knowles et al. 2023). It is possible that a parasite with similar phenology eluded our collection during mid‐summer (August) in North America.

Despite similarities of the shell anomaly described in the northeastern United States (Strayer 2008; present study) to that caused by *X. canterburyensis* in New Zealand (Forsyth and McCallum 1978; Forsyth 1979), a parasite has not been identified in the northeastern United States. The deformity appears to differ in description from those previously reported from the region (Beecher 1883), possibly suggesting that this is a relatively new phenomenon in the region (Strayer 2008), and neither a contaminant nor a recent range expansion of a parasite can be ruled out as a causative agent.

The effects of this deformity on individual fitness or mussel population growth are unclear, but we did collect younger normal animals, which suggests possible recruitment at the site in Massachusetts. Comparative investigations of mussel longevity, fecundity, and recruitment rates between populations with and without the deformity are warranted to fully understand the effects of the deformity. We also believe that combined evaluations of environmental pollutants and parasite occurrence are needed to better understand the extent and effect of this deformity. Furthermore, the overwhelming decline in native unionid diversity warrants further investigation into shell deformities and mussel parasites as potential metrics of mussel health.

## AUTHOR CONTRIBUTIONS

P.D.H., A.G.L., and A.M. were responsible for the concept and experimental design; P.D.H., A.G.L., A.M., and K.O. performed data collection; P.D.H., A.G.L., and K.O. conducted data analysis; and P.D.H., A.G.L., A.M., K.O., and J.C. wrote and edited the manuscript.

## CONFLICT OF INTEREST STATEMENT

The authors claim no conflicts of interest.

## ETHICS STATEMENT

All research described in this paper was conducted under collection permits from the MDFW.

## Supporting information


Figure S1:

## Data Availability

Data that support the findings of this study are available from the corresponding author upon reasonable request. Data describing the locations of sensitive species are not available without confirmed consent from the appropriate state fish and wildlife agencies.
