# Supplementary figures and images for: Description and potential sources of a shell deformity in North American freshwater mussels (Unionoida)
Source: J Aquat Anim Health. 2024 Dec 1;36(4):310–20. doi: 10.1002/aah.10232 (PMC11685056; doi:10.1002/aah.10232)

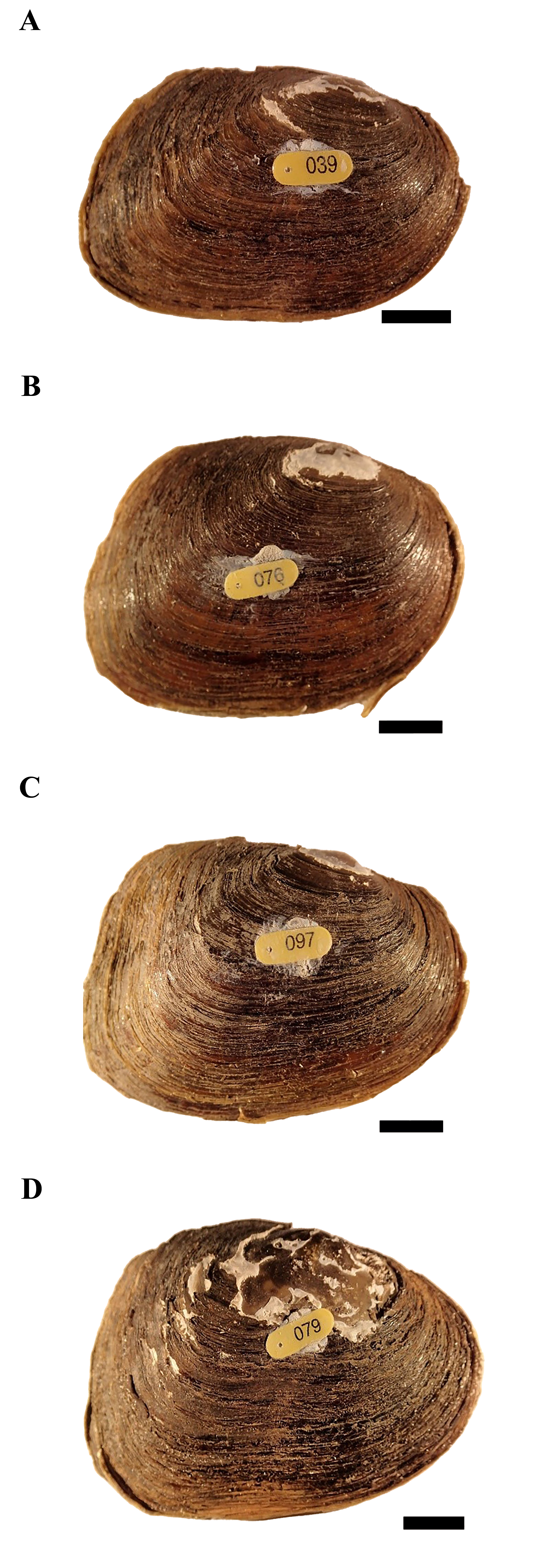

Supplement: Supplementary file 1 — Figure S1: [file AAH-36-310-s001.png]
